# Sperm Quality and Fertility of Livestock Animals

**DOI:** 10.3390/ani13040604

**Published:** 2023-02-09

**Authors:** Jordi Ribas-Maynou, Isabel Barranco, Albert Salas-Huetos

**Affiliations:** 1 Unit of Cell Biology, Department of Biology, Faculty of Sciences, University of Girona, ES-17003 Girona, Spain; 2 Biotechnology of Animal and Human Reproduction (TechnoSperm), Institute of Food and Agricultural Technology, University of Girona, ES-17003 Girona, Spain; 3 Department of Veterinary Medical Sciences, University of Bologna, Ozzano dell’Emilia, 40126 Bologna, Italy

Recent research has focused on the understanding of the causes of subfertility observed in livestock species, evidencing that different factors could underlie this condition [1,2]. Among these causes, and taking into account that embryos are formed by the fusion of an oocyte with a sperm cell, it is obvious that good gamete quality is one of the most important features to generate a healthy embryo which develops into a viable offspring. In this regard, research into livestock fertility has focused greatly on the study of male factor, having developed not only strategies for the preservation of seminal doses [3], but also a diverse panel of biomarkers to identify and select the best animals to become semen producers and predict their future reproductive efficiency. Despite these advances, investigations in the present Special Issue have been able to uncover new biomarkers and factors affecting sperm quality, which could improve our understanding of livestock fertility.

Firstly, in “ProAKAP4 Concentration Is Related to Sperm Motility and Motile Sperm Subpopulations in Frozen–Thawed Horse Semen”, Dordas-Perpinyà et al. have shown that the levels of proAKAP4 in semen are related to the ability of sperm cells to maintain flagellum functionality in stallions [4]. In this study, the authors analyzed sperm motility through computer-assisted sperm analysis and proAKAP4 levels through ELISA in 48 different ejaculates from 13 different stallions. The results showed that total motility was lower in ejaculates with low levels of proAKAP4, and that the slowest of the four different sperm subpopulations identified in terms of motility presented an association with lower levels of proAKAP4. In conclusion, the authors suggest that high levels of proAKAP4 are associated with stallion sperm motility; however, further studies are needed to establish proAKAP4 as a sperm motility biomarker in stallions.

Secondly, in “Effect of Epidermal Growth Factor (EGF) on Cryopreserved Piedmontese Bull Semen Characteristics”, Alkhawagah et al. investigated whether a supplementation with different concentrations of EGF of a bull extender prior to freezing could help to maintain sperm quality after thawing [5]. The study assessed different concentrations of EGF (0, 100, 200 and 400 ng/mL) in pooled samples from four bulls collected over an 8-week period, and evaluated sperm motility, viability, acrosomal integrity, DNA integrity, mitochondrial activity, antioxidant activity and other functional parameters, such as the sperm mucus penetration test and fertilizing ability. The results showed that supplementation with the two highest doses had a positive effect on the maintenance of sperm motility after thawing, but no differences were observed regarding fertilizing ability.

In the study by Schulze and Waberski, entitled “Compensability of Enhanced Cytoplasmic Droplet Rates in Boar Semen: Insights of a Retrospective Field Study”, the authors evaluated whether the incidence of cytoplasmic droplets, a common abnormality found in boar sperm, have a negative incidence in fertility outcomes [6]. In this retrospective study, they included 260 boar ejaculates used for 1947 inseminations, generating four groups with different incidence of cytoplasmic droplets. The results evaluated whether the four cytoplasmic droplet incidence groups presented differences in sperm count, membrane integrity and fertility records, concluding that when cytoplasmic droplets exceeded 11%, lower litter sizes were obtained.

In “Environmental Factors Affecting the Reproductive Efficiency of Italian Simmental Young Bulls”, Corte Pause et al. aimed to study the effect of weather conditions and scrotal circumference on fresh semen characteristics from 577 bulls [7]. In their study, evidence is shown that scrotal circumference was related to sperm count, that the temperature and humidity sixty days before collection was associated with sperm morphology abnormalities, and that the temperature and humidity thirty days and ten days before ejaculation influence sperm motility. Therefore, the authors conclude that environmental conditions are essential to establish the best time-frame to obtain semen samples from bulls.

Koch et al., in their study “Effect of Different Thawing Methods for Frozen Bull Semen and Additional Factors on the Conception Rate of Dairy Cows in Artificial Insemination”, aimed to evaluate whether different thawing methods of bull semen influence the success of artificial insemination in dairy cows [8]. In their study, they tested three protocols: 11 s at 38 °C in a water bath, 35 s at 38 °C in a water bath, and 30 s “in the cow”. No differences in the conception rate were observed for the three treatments, while they observed that other factors such as the lactation number, the month of insemination, or the insemination method could be influencing the conception rates. On the basis of their results, the authors stated that all methods are useful for bull sperm thawing, presenting a similar efficiency.

Next, in “Dominant Components of the Giant Panda Seminal Plasma Metabolome, Characterized by 1H-NMR Spectroscopy”, Zhu et al. focused on characterizing the metabolome of the giant panda seminal plasma, in order to find biomarkers associated with the breed in this species [9]. The authors obtained semen samples from 15 animals using electroejaculation and used them to describe the metabolic profile of giant panda seminal plasma. Out of the 35 metabolites identified, they could identify a specific metabolome profile for the three age groups established (<8 years old, between 8 and 12 years old, and >13 years old), and showed that seminal plasma metabolome is related to estrus phases throughout the year. The results obtained shed light on the breeding approach for giant pandas.

Quiñones-Perez et al., in their study “The Semen Microbiome and Semen Parameters in Healthy Stallions”, addressed the limited information that is present regarding the microflora in stallion ejaculates [10] and its association with sperm quality parameters. The study included semen samples from 12 stallions aged 6 to 23 years old, in which sperm quality was assessed through concentration, motility and DNA damage, and the microbiome was assessed through 16S rRNA sequencing. The results identified two bacterial families (Peptoniphilaceae and Clostridiales Incertae Sedis XI) that were correlated with sperm motility, and despite the interactions between bacteria and spermatozoa needing to be further elucidated, these bacterial families can be used as potential biomarkers for sperm quality.

In the study “Protein Identification of Seminal Plasma in Bali Bull (Bos javanicus)”, Iskandar et al. assessed the seminal plasma proteome of Bali bull in search of biomarkers for fertility. In their study, semen samples from 10 animals aged 5 to 10 years were used, and 94 proteins were identified through Liquid Cromatography and Mass Spectrometry. Among these proteins, six proteins were related to fertility status, becoming putative fertility biomarkers for this species.

Finally, in the study “Telomere Length in Pig Sperm Is Related to In Vitro Embryo Development Outcomes”, Ribas-Maynou et al. estimated pig sperm telomere length for the first time and assessed its relation to in vitro embryo development outcomes [11]. In this work, they conducted IVF using semen samples from 23 different Piétrain boars and identified a significant correlation between sperm telomere length and the percentage of morulae 6 days after IVF. Therefore, in light of these results, the authors suggested that sperm telomere length can be used as a biomarker for embryo development.

As the scientific community will be able to read in the present Special Issue, the range of topics addressed by the authors show the diverse horizons to approaching animal reproduction. In general, the papers included in the *Animals* Special Issue entitled “Sperm Quality and Fertility of Livestock Animals” provide new biomarkers associated with reproductive success, and identify different factors involved in artificial insemination in livestock species.
